# OsWRKY67 Plays a Positive Role in Basal and XA21-Mediated Resistance in Rice

**DOI:** 10.3389/fpls.2017.02220

**Published:** 2018-01-11

**Authors:** Kieu T. X. Vo, Chi-Yeol Kim, Trung V. Hoang, Sang-Kyu Lee, Gautam Shirsekar, Young-Su Seo, Sang-Won Lee, Guo-Liang Wang, Jong-Seong Jeon

**Affiliations:** ^1^Graduate School of Biotechnology and Crop Biotech Institute, Kyung Hee University, Yongin, South Korea; ^2^Department of Plant Pathology, The Ohio State University, Columbus, OH, United States; ^3^Department of Microbiology, Pusan National University, Busan, South Korea

**Keywords:** disease resistance, *Magnaporthe oryzae*, OsWRKY67, rice, *Xanthomonas oryzae* pv. *oryzae*

## Abstract

WRKY proteins play important roles in transcriptional reprogramming in plants in response to various stresses including pathogen attack. In this study, we functionally characterized a rice WRKY gene, *OsWRKY67*, whose expression is upregulated against pathogen challenges. Activation of *OsWRKY67* by T-DNA tagging significantly improved the resistance against two rice pathogens, *Magnaporthe oryzae* and *Xanthomonas oryzae* pv. *oryzae* (*Xoo*). Reactive oxygen species (ROS) rapidly accumulated in *OsWRKY67* activation mutant lines in response to elicitor treatment, compared with the controls. Overexpression of *OsWRKY67* in rice confirmed enhanced disease resistance, but led to a restriction of plant growth in transgenic lines with high levels of OsWRKY67 protein. *OsWRKY67* RNAi lines significantly reduced resistance to *M. oryzae* and *Xoo* isolates tested, and abolished XA21-mediated resistance, implying the possibility of broad-spectrum resistance from OsWRKY67. Transcriptional activity and subcellular localization assays indicated that OsWRKY67 is present in the nucleus where it functions as a transcriptional activator. Quantitative PCR revealed that the pathogenesis-related genes, *PR1a, PR1b, PR4, PR10a*, and *PR10b*, are upregulated in *OsWRKY67* overexpression lines. Therefore, these results suggest that OsWRKY67 positively regulates basal and XA21-mediated resistance, and is a promising candidate for genetic improvement of disease resistance in rice.

## Introduction

The WRKY proteins possess a unique conserved domain, WRKYGQK, and can bind to the DNA sequence (T)(T)TGAC(C/T) called the W-box (Eulgem et al., 2000). A number of studies suggest that WRKYs play a key role in transcriptional reprogramming upon pathogen perception, as either an activator or a repressor (Pandey and Somssich, 2009; Rushton et al., 2010; Chen et al., 2012). WRKYs regulate expression of pathogenesis-related (PR) genes (Jimmy and Babu, 2015) and induce defense metabolism pathways (Jiang et al., 2017) directly or indirectly; thus, they are a key family for molecular studies, as well as breeding programs. Since its first cloning in sweet potato, WRKY genes have been identified in numerous plant species, including the cereal model crop rice (Wu et al., 2005; Huang et al., 2012; Rinerson et al., 2015). In rice, for instance, data accumulated from microarray analysis and RNA sequencing revealed that OsWRKYs dynamically respond to various stimuli, including biotic and abiotic stresses (Ramamoorthy et al., 2008). A number of OsWRKY genes are induced by *Magnaporthe oryzae* and *Xanthomonas oryzae* pv. *oryzae* (*Xoo*), the causal agents of rice blast and bacterial leaf blight disease, respectively (Ryu et al., 2006).

Plants have complicated innate immune systems, including both PAMP-triggered immunity (PTI) and effector-triggered immunity (ETI) (Dangl and Jones, 2001; Jones and Dangl, 2006). The defense mechanisms activate a series of physiological and biochemical processes, including modulation of phytohormones, accumulation of reactive oxygen species (ROS), and expression of PR genes (Schwessinger and Zipfel, 2008; Tena et al., 2011). In rice, the reverse genetics approach has deciphered the involvement of many OsWRKYs in defense mechanisms. For instance, OsWRKY45, which is activated by benzothiadiazole (BTH), plays a crucial role in salicylic acid (SA)-inducible resistance to rice blast and bacterial leaf blight diseases by priming diterpenoid phytoalexin biosynthesis (Shimono et al., 2007, 2012; Akagi et al., 2014). Regulation of OsWRKY45 is controlled by mitogen-activated protein kinase (MAPK)-dependent phosphorylation, and the ubiquitin–proteasome system (Matsushita et al., 2013; Ueno et al., 2013). OsWRKY42 is a negative regulator of the defense response to *M. oryzae*, and works by suppressing jasmonic acid (JA) signaling; OsWRKY42, in turn, is suppressed by OsWRKY13, a target of OsWRKY45 (Cheng et al., 2015). OsWRKY33 directly binds to the promoter of a number of defense-related genes (Koo et al., 2009). The rice homolog of *Arabidopsis Nonexpressor of PR genes 1* (*AtNPR1*), NH1, positively controls blast resistance (Chern et al., 2005) and was found to be downstream of OsWRKY12 (Liu et al., 2005). PR genes and defense-related genes are upregulated by enhanced expression of several OsWRKYs—*OsWRKY6, OsWRKY12, OsWRKY22, OsWRKY31, OsWRKY53, OsWRKY71*, and *OsWRKY77* (Jimmy and Babu, 2015).

WRKY genes have been examined for genetic improvement of defense mechanisms of crops against pathogens via either overexpression or suppression. For instance, in rice, resistance against diseases caused by *M. oryzae* and *Xoo* is gained by overexpression of *OsWRKY13, OsWRKY30*, and *OsWRKY45*. While the overexpression of *OsWRKY31, OsWRKY22*, and *OsWRKY53* increases resistance against fungal rice blast, the ubiquitous expression of *OsWRKY51* and *OsWRKY71* has a beneficial effect on resistance to bacterial leaf blight disease (Pandey and Somssich, 2009; Peng et al., 2012; Hwang et al., 2016). Recently, the positive regulation of resistance to *Rhizoctonia solani* by the module of *OsWRKY80* and *OsWRKY4* was observed by the ectopic expression of *OsWRKY80* (Peng et al., 2016).

The rice XA21 confers robust resistance to bacterial blight disease by recognizing the extracellular signal molecule from *Xoo* (Song et al., 1995). Functional XA21 requires a number of association proteins, such as an endoplasmic reticulum chaperone (OsBiP3), the rice somatic embryogenesis receptor kinase 2 (OsSERK2), and other XA21-binding proteins (XBs) such as XB3 (an E3 ubiquitin ligase), XB10 (OsWRKY62), XB15 (a PP2C phosphatase), and XB24 (an ATPase) (Liu et al., 2014). Previously, OsWRKY62, OsWRKY76, and OsWRKY28 were reported to be negative regulators of XA21-mediated resistance by suppressing the activation of defense-related genes *PR1* and *PR10* (Peng et al., 2008; Yokotani et al., 2013).

In this study, we functionally characterized a rice WRKY gene, *OsWRKY67*, whose expression is upregulated against *M. oryzae* and *Xoo* challenges. Through analysis of its mutant and transgenic rice lines, we demonstrated that a modulated expression of *OsWRKY67* alters the defense response to rice blast fungus and bacterial blight, as well as plant growth. Moreover, we also showed that OsWRKY67 is required for XA21-mediated resistance.

## Materials and methods

### Plant materials and growth

The *OsWRKY67* T-DNA activation tagging mutant lines (*OsW67-D*) and *OsWRKY67* overexpression lines (*OsW67-OX*) with a background wild type japonica cultivar, Dongjin (DJ), and *OsWRKY67* RNAi lines (*OsW67-RNAi*) with a background transformation cultivar, Kitaake, expressing *Xa21* (Kit-XA21) (Park et al., 2010), were used in this study. Rice plants were grown in a greenhouse at 30°C during the day and at 20°C at night, under a light/dark cycle of 14/10 h with a photosynthetic photon flux density of 1,800 μmol m^−2^ s^−1^, and at a relative humidity of 60%.

### Pathogen inoculation

To examine the defense response to pathogen infection, *M. oryzae* isolates PO6-6 and RO1-1 were cultured on V8 juice agar plates [80 mL V8 Juice (Campbell's Soup Company, Camden, NJ) l^−1^, 15 g agar l^−1^, pH 6.8] for 2 weeks under fluorescent light. Subsequently, spores were collected and suspended in water to reach a concentration of 5 × 10^6^ ml^−1^. Fully expanded leaves of 6-week-old plants were inoculated following the spot inoculation method described previously (Kanzaki et al., 2002). Leaf samples were collected to evaluate the lengths of disease lesions, which were characterized by browned areas, and were photographed 9 days post-inoculation (DPI).

For bacterial inoculation, *Xoo* isolates PXO99 and KXO85 (Strain number KACC10331) were first cultured on peptone sucrose agar plates (sucrose 10 g l^−1^, peptone 10 g l^−1^, Na-glutamate 1 g l^−1^, agar 15 g l^−1^, pH 6.8), and then suspended in water using the leaf clipping method (Han et al., 2013). Six-week-old plants were used for inoculation. The lesion length at 12 DPI was defined by the water-soak lesion, indicating successful infection by *Xoo* (Han et al., 2013). Three biological replicates were tested in each experiment for pathogen inoculations. Student's *t*-test was used to show statistical differences.

### Identification of activation tagging T-DNA mutants

Two *OsWRKY67* activation mutants, *OsW67-D1* (Line number 3A-51109) and *OsW67-D2* (Line number 3A-02704), with the 4x CaMV35S (35S) promoter enhancer element insertion approximately 7 and 4 kb upstream of the *OsWRKY67* coding sequence, respectively, were isolated from the T-DNA insertion sequence database (Jeong et al., 2002). Homozygous mutants for T-DNA insertion were identified by genomic DNA PCR with OsW67-D1/D2-F/R and T-DNA-specific (2715LB) primers (Table S1). PCR amplification was performed in a final volume of 40 μl [100 pmol each primer, 20 μM each dNTPs, 10 mM Tris–HCl (pH 9.0), 2 mM MgCl_2_, 50 mM KCl, 0.1% Triton X-100, and 0.5 U Taq polymerase] including 50 ng genomic DNA as a template. The amplification conditions were: 94°C for 5 min, followed by 35 cycles of 94°C for 1 min; 58°C for 1 min; and 72°C for 1 min, with a final extension at 72°C for 5 min.

### Measurement of ROS

ROS generation after elicitor (100 nM flg22, 8 nM hexa-N-acetyl-chitohexaose, or water as a control) treatment was measured using the luminol chemiluminescence assay with leaf disks from 4-week-old plants, as described previously (Park et al., 2012). Following the treatment, luminescence was measured at 10 s intervals with a Glomax 20/20 luminometer (Promega, Madison, WI). Three replications were carried out for each sample. Student's *t*-test was used to show statistical differences.

### Sheath inoculation assay

Penetration assays were performed using rice leaf sheaths. For the leaf sheath assay, a conidial suspension (2 × 10^4^ conidia ml^−1^) of *M. oryzae* PO6-6 was injected into excised rice sheaths and incubated in a moistened box at room temperature for 48 h (Koga et al., 2004). The infected rice sheaths were then trimmed to remove chlorophyll-enriched parts. Epidermal layers of the mid vein (three to four cell layers thick) were used for clear microscopic observation of penetration and infectious growth.

### Production of *35S:OsWRKY67* and RNAi transgenic rice plants

The full-length *OsWRKY67* cDNA was amplified from total RNA of the leaves of 3-week-old rice plants by RT-PCR, using the OsW67-F/R primers encompassing the translation start and stop codons (Table S1). The PCR product was subcloned into the pGEM T-easy vector (Promega) and confirmed by sequencing. The *OsWRKY67* cDNA was digested with *Bam*HI and *Xho*I and then cloned between the 35S promoter and four sequences of c-Myc tags, followed by Nos terminator of the plant expression vector pJJ1754 containing the hygromycin resistance gene as a selection marker. To construct the plasmid for RNAi, a unique 359 bp sequence of *OsWRKY67* was amplified by PCR with OsW67-RNAi-F/R primers, and inserted into the pANDA vector with the antisense sequence upstream of the sense sequence as described (Miki and Shimamoto, 2004).

To produce transgenic rice plants expressing *35S*:*OsWRKY67* in the background cultivar, Dongjin, and *OsWRKY67* RNAi in the background Kitaake carrying *Xa21* (Park et al., 2010), *Agrobacterium tumefaciens* LBA4404 strains harboring these vectors were grown on AB media (K_2_HPO_4_ 3 g l^−1^, NaH_2_PO_4_ 1 g l^−1^, NH_4_Cl 1 g l^−1^, MgSO_4_ 0.3 g l^−1^, KCl 0.15 g l^−1^, CaCl_2_ 7.5 mg l^−1^, FeSO_4_ 2.5 mg l^−1^) supplemented with 10 mg l^−1^ kanamycin for 3 days at 28°C; transgenic calli were obtained via the *Agrobacterium*-mediated co-cultivation method as described previously (Jeon et al., 2000). Transgenic rice plants were selected on a medium containing 50 mg l^−1^ hygromycin and 250 mg l^−1^ cefotaxime.

### Quantitative PCR, western blot analysis, and phosphatase treatment

In order to analyze the level of transcriptional expression of *OsWRKY67* and PR genes in the *OsWRKY67* activation mutants, RNAi transgenic plants, and overexpression lines, total RNA was extracted using RNAiso Plus according to the manufacturer's protocol (Takara Bio, Kyoto, Japan). Reverse transcription was performed according to the manufacturer's protocol using ReverTra Ace® qPCR RT Master Mix with gDNA Remover (Toyobo, Osaka, Japan). Quantitative PCR (qPCR) was performed using Qiagen 154 Rotor-Gene Q real-time PCR cycler with thermal cycling procedure: 95°C for 40 s, 57°C for 40 s, and 72°C for 40 s. The rice gene *Ubiquitin5* (*OsUbi5*; LOC_Os01g22490) (Jain et al., 2006) was used as an endogenous control to normalize variance in the quality of RNA and the amount of cDNA. All of the primers for qPCR analyses are summarized in Table S1.

To quantify protein levels of OsWRKY67 in *OsW67-OX* transgenic rice lines, total protein from leaf samples was extracted in 50 mM Tris-HCl (pH 7.5), 150 mM NaCl, 1 mM EDTA, 0.5% NP-40, loaded onto a 12% SDS-PAGE gel, and blotted onto a nitrocellulose membrane. The membrane was incubated overnight at 1:5,000 anti-c-Myc antibody (Bethyl Laboratories, Montgomery, TX), washed three times with tris-buffered saline (TBST; pH 7.4, 0.1% Tween 20), incubated with horseradish peroxidase (HRP)-conjugated secondary antibody (Bethyl Laboratories), and washed three times with TBST. The membrane was then developed with an enhanced chemiluminescence (ECL) detection system (LI-COR Biosciences, Lincoln, NE).

To examine the phosphorylation of OsWRKY67, 40 μg of total protein extracted from *OsWRKY67* overexpression lines was incubated with 1 μl of lambda phosphatase for 30 min at 30°C (Matsushita et al., 2013), in accordance with the manufacturer's protocol (400 U μl^−1^, P0753S, New England Biolabs, Ipswich, MA). After adding 5x SDS sample buffer, the samples were boiled for 5 min at 95°C.

### Subcellular localization of OsWRKY67-GFP fusion protein

The full-length cDNA lacking the stop codon of *OsWRKY67* was cloned into the pENTR^TM^/D-TOPO vector (Invitrogen, Gaithersburg, MD). The insert as confirmed by sequencing was cloned into the destination vector p2GWF7 (Karimi et al., 2002) for C-terminal GFP (Green Fluorescent Protein) fusion using the Gateway LR clonase (Invitrogen, Gaithersburg, MD). The construct was introduced into the protoplasts of maize mesophyll, using the PEG-calcium mediated transformation method (Cho et al., 2009). OsABF1-RFP (Red Fluorescent Protein) was used as a nuclear marker (Hossain et al., 2010). Expression of the OsWRKY67-GFP was monitored at 12–24 h incubation to allow transient expression using a confocal microscope (LSM510 META, Carl Zeiss, Jena, Germany). The GFP fluorescence was excited at 488 nm and detected in the emission range of 500–525 nm.

### Transcriptional activation assay

The effector vector for the transcriptional activation assay was constructed by ligating the *OsWRKY67* cDNA containing *Sma*I and *Sal*I restriction sites that were obtained by PCR using OsW67-Smal-F/SalI-R primers (Table S1); the resulting vector contained the 35S promoter, the tobacco mosaic virus (TMV) translation enhancer (Ω) sequence, and the *OsWRKY67* cDNA insert fused to the GAL4 DNA binding domain (BD) in frame. The GAL4-responsive reporter vector contained 5X GAL4, minimal TATA, the Ω sequence, and the *Luciferase (LUC)* gene (Ohta et al., 2000). The maize *Ubiquitin1* promoter:β*-glucuronidase* (*ZmUBQ1:GUS*) construct was used as an internal control. Maize mesophyll protoplasts isolated from the second leaves of dark grown plants were cotransfected with the effector, reporter, and internal control vectors as described previously (Cho et al., 2009).

To test transcriptional activation ability in yeast, full-length cDNA of *OsWRKY67* with *Eco*RI/*Bam*HI sites amplified by PCR with OsW67-YF/YR primers (Table S1), was cloned into pGBKT7. The junction between the GAL4 DNA binding domain and *OsWRKY67* was confirmed by DNA sequencing. The bait plasmid was introduced with an empty prey vector into a yeast strain PBN204. The negative control prey vector was pACT2, which contained a GAL4 transcriptional activation domain. PBN204 had *ADE2, URA3*, and *lacZ* genes as reporters. Yeast transformants were selected on an SD minimal medium lacking leucine and tryptophan (SD-LW). The colonies were then replica-plated on various SD selection media, such as a medium lacking leucine, tryptophan, and uracil (SD-LWU), and a medium lacking leucine, tryptophan, and adenine (SD-LWA), and used to assess β-galactosidase activity in an X-gal filter assay.

## Results

### Evaluation of disease resistance of *OsWRKY67* activation mutants to rice pathogens

**Figure 1:**
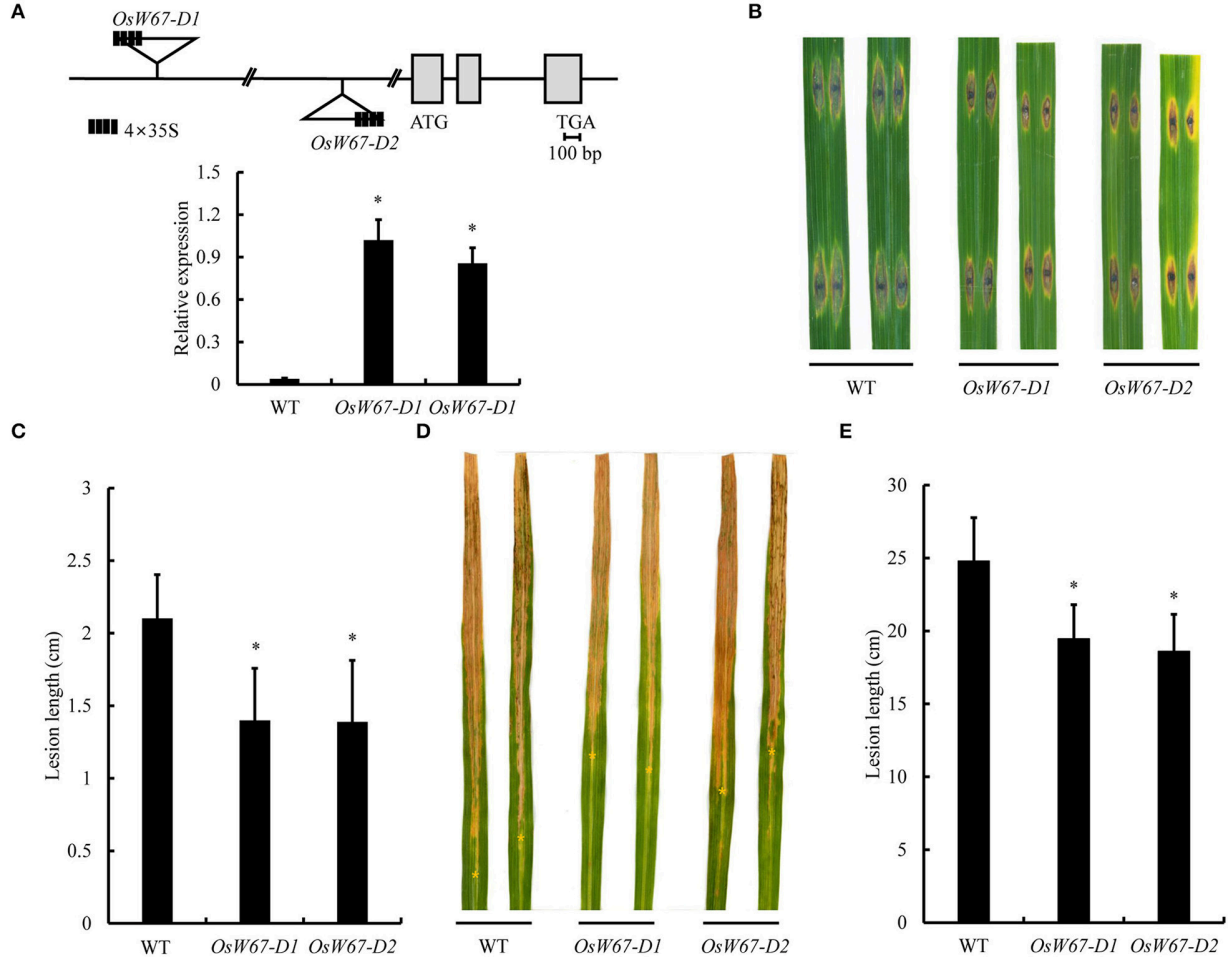
Disease resistance phenotype of *OsWRKY67* activation mutants. **(A)** Schematic representation of *OsWRKY67* activation mutant alleles *OsW67-D1* and *OsW67-D2* with T-DNA insertion carrying a 4 × 35S enhancer on the diagram of the *OsWRKY67* gene. Gray boxes and black lines represent exons and introns, respectively (Top). Levels of *OsWRKY67* expression were validated by qPCR (Bottom). WT is a wild type control cultivar, Dongjin. *OsUbq5* is a PCR control. **(B)** Representative leaves 9 days after inoculation with *M. oryzae* PO6-6. **(C)** Disease lesion lengths measured 9 days after inoculation with *M. oryzae* PO6-6. **(D)** Representative leaves 12 days after inoculation with *Xoo* PXO99. Yellow asterisks indicate bottoms of lesions. **(E)** Disease lesion lengths measured 12 days after inoculation with *Xoo* PXO99. Data are represented as means ± SD. Asterisks represent statistical significance with Student's *t*-test, *p* < 0.0001.

Our previous observation of upregulation of *OsWRKY67* in response to invading *M. oryzae* and *Xoo* pathogens (Ryu et al., 2006) suggests its involvement in disease defense. To elucidate OsWRKY67 function, two T-DNA activation tagging mutant plants with enhanced expression of *OsWRKY67* were isolated from our T-DNA activation library and named *OsW67-D1* and *OsW67-D2* (Figure 1A, Top) (Jeon et al., 2000; Jeong et al., 2002). The level of the *OsWRKY67* transcript was examined by qPCR in the two activation mutant alleles (Figure 1A, Bottom). The result revealed a substantial increase in gene expression by the enhancer element that was present in the introduced T-DNA. The homozygous mutant plants were isolated by genomic DNA PCR (using T-DNA- and gene-specific primers) from segregating progeny plants, and challenged with the pathogens, *M. oryzae* and *Xoo*. A severe disease symptom featured by brown lesions arose from the compatible interactions in wild type control, Dongjin (Figures 1B–E). In contrast, *OsW67-D1* and *OsW67-D2* plants exhibited significantly reduced disease lesions when challenged with virulent pathogens *M. oryzae* PO6-6 (Figures 1B,C) and *Xoo* PXO99 (Figures 1D,E). The enhanced disease resistance phenotype was confirmed in the activation mutant lines in the following generations, which supported stable inheritance of the trait in *OsW67-D1* and *OsW67-D2* lines.

### ROS production in *OsWRKY67* activation mutants in response to elicitor treatment

**Figure 2:**
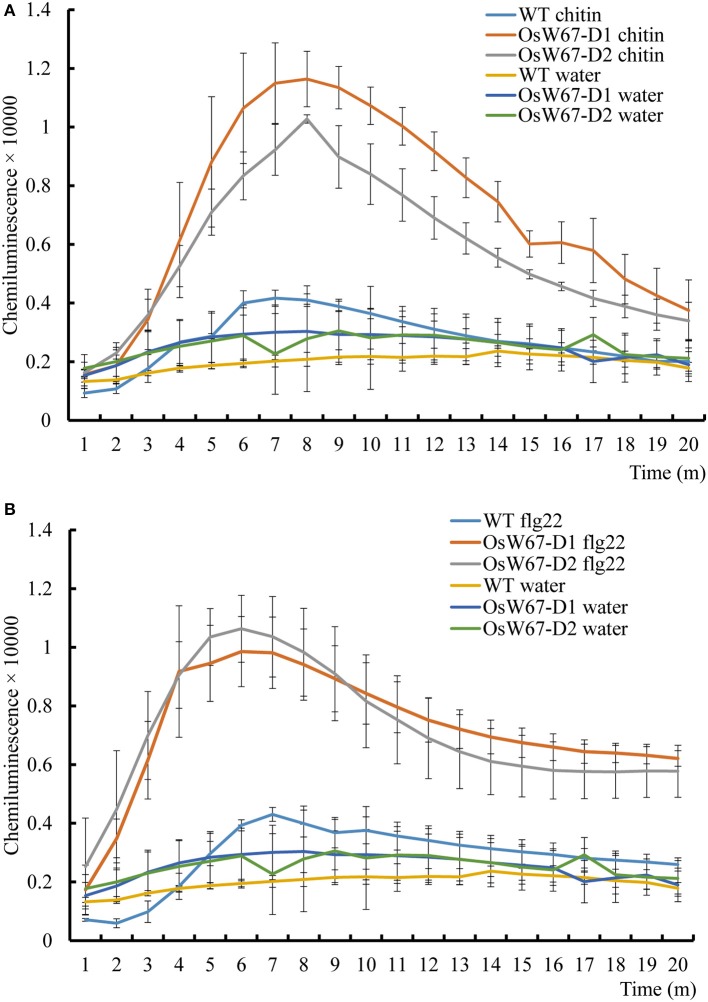
ROS production of *OsWRKY67* activation mutants in response to elicitor treatments. **(A)** A chitin-induced ROS burst in the control WT and *OsW67-D* mutants. Rice leaf disks were treated with 8 nM chitin (hexa-*N*-acetyl-chitohexaose) and water. WT is a wild type control cultivar, Dongjin. **(B)** A flg22-induced ROS burst in the control and *OsW67-D* mutants. Rice leaf disks were treated with 100 nM flg22 and water. ROS were detected with a luminol-chemiluminescence assay. Error bars represents the SE (*n* = 3).

ROS are potent signal molecules that are rapidly generated in response to stress, and are a common feature of the plant immune response (Stael et al., 2015). In *Arabidopsis* and rice, flg22 and chitin have been shown to trigger ROS production (Mersmann et al., 2010; Petutschnig et al., 2010; Park et al., 2012). In our experiments, *OsW67-D* activation mutant plants significantly induced ROS just 3 min after treatment with flg22 and chitin elicitation, compared to the wild type control and water treatment. ROS production kept increasing and reached a peak that was more than 2-fold greater than the control at 8 min in the case of chitin (Figure 2A), and at 6 min in the case of flg22 (Figure 2B). Meanwhile, the wild type control and all water-treated samples displayed a steady level of ROS. Together, these data suggest an enhanced basal resistance capacity of the *OsW67-D* mutant lines.

### Disease resistance of *OsWRKY67* overexpression lines

**Figure 3:**
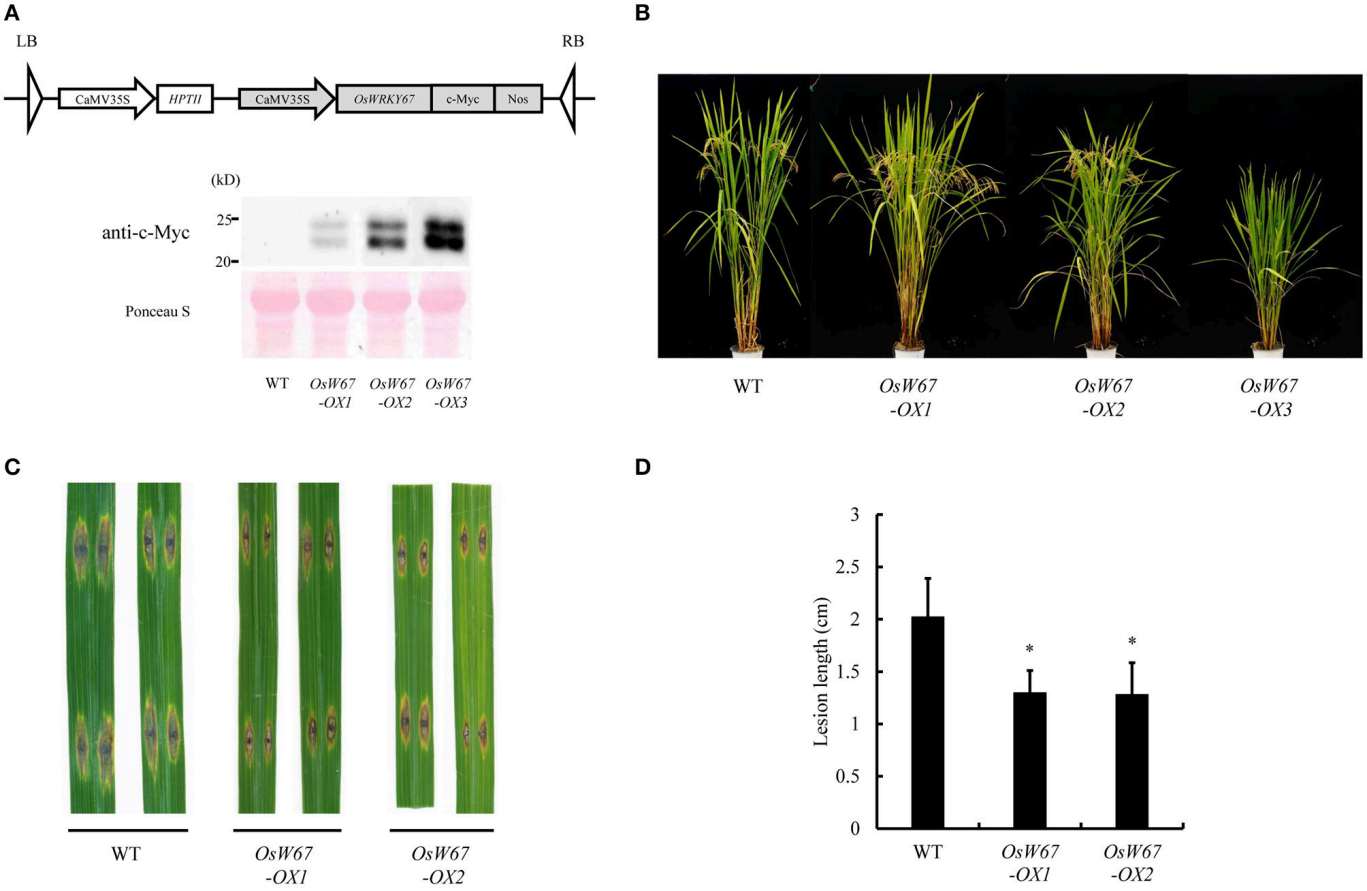
Disease resistance phenotype of *OsWRKY67* overexpression lines. **(A)** Schematic representation of the *OsWRKY67* overexpression construct (Top). Western blot analysis using the anti-c-Myc antibody to examine OsWRKY67 protein (Bottom). Ponceau S staining was used as the loading control. **(B)** Growth phenotype of *OsWRKY67* overexpression lines. Rice plants were grown in a paddy field and photographed at the ripening stage. WT is a wild type control cultivar, Dongjin. **(C)** Representative leaves 9 days after inoculation with *M. oryzae* PO6-6. **(D)** Disease lesion lengths measured 9 days after inoculation with *M. oryzae* PO6-6. Data are represented as means ± SD. Asterisks represent statistical significance with Student's *t*-test, *p* < 0.0001.

Defense response is a sudden process that consumes a lot of energy and results in many changes within the cell and the whole plant. Thus, employing resistance regulators in breeding usually causes autoimmune-like fitness penalties (Delteil et al., 2010). To validate the effect of an enhanced level of *OsWRKY67* expression in rice, the overexpression construct that produces OsWRKY67 fused with the c-Myc peptide at the C-terminus driven by the 35S promoter, was introduced into rice by *Agrobacterium* mediation (Figure 3A, Top). The expression of OsWRKY67 protein in each individual plant was examined by Western blot analysis using the anti-c-Myc antibody. Two c-Myc fusion bands were detected in the three independent lines with different expression levels, in which the lower reflects the predicted size of 21 kD of the OsWRKY67-Myc protein (Figure 3A, Bottom). OsWRKY67 is classified into the group Ib, in which many of these proteins undergo phosphorylation as a post-translational modification process (Wu et al., 2005). We treated the proteins extracted from *OsWRKY67*-overexpressing leaves with a lambda phosphatase to test whether OsWRKY67 was phosphorylated. However, the two bands detected were retained after the treatment (Figure S1), suggesting that OsWRKY67 is not likely a target of phosphorylation.

Among the independent overexpression lines obtained, strong expressors (for example, *OsW67-OX3*) with a very high accumulation of OsWRKY67 exhibited severe suppression of plant growth and sterility (Figure 3B). In contrast, the weak expressors (for example, *OsW67-OX1*) displayed little or no growth penalty, which indicates that the exaggerated level of OsWRKY67 caused growth retardation. Two lines with normal seed sets, *OsW67-OX1* and *OsW67-OX2*, were challenged with *M. oryzae* PO6-6. *OsW67-OX* lines displayed a significantly reduced disease phenotype, while the wild type control developed severe disease (Figures 3C,D). This result clearly confirmed the enhanced resistance of activation tagging mutants of *OsWRKY67, OsW67-D1*, and *OsW67-D2*, to pathogens.

### Analysis of disease resistance in leaf sheath cells with increased expression of *OsWRKY67*

**Figure 4:**
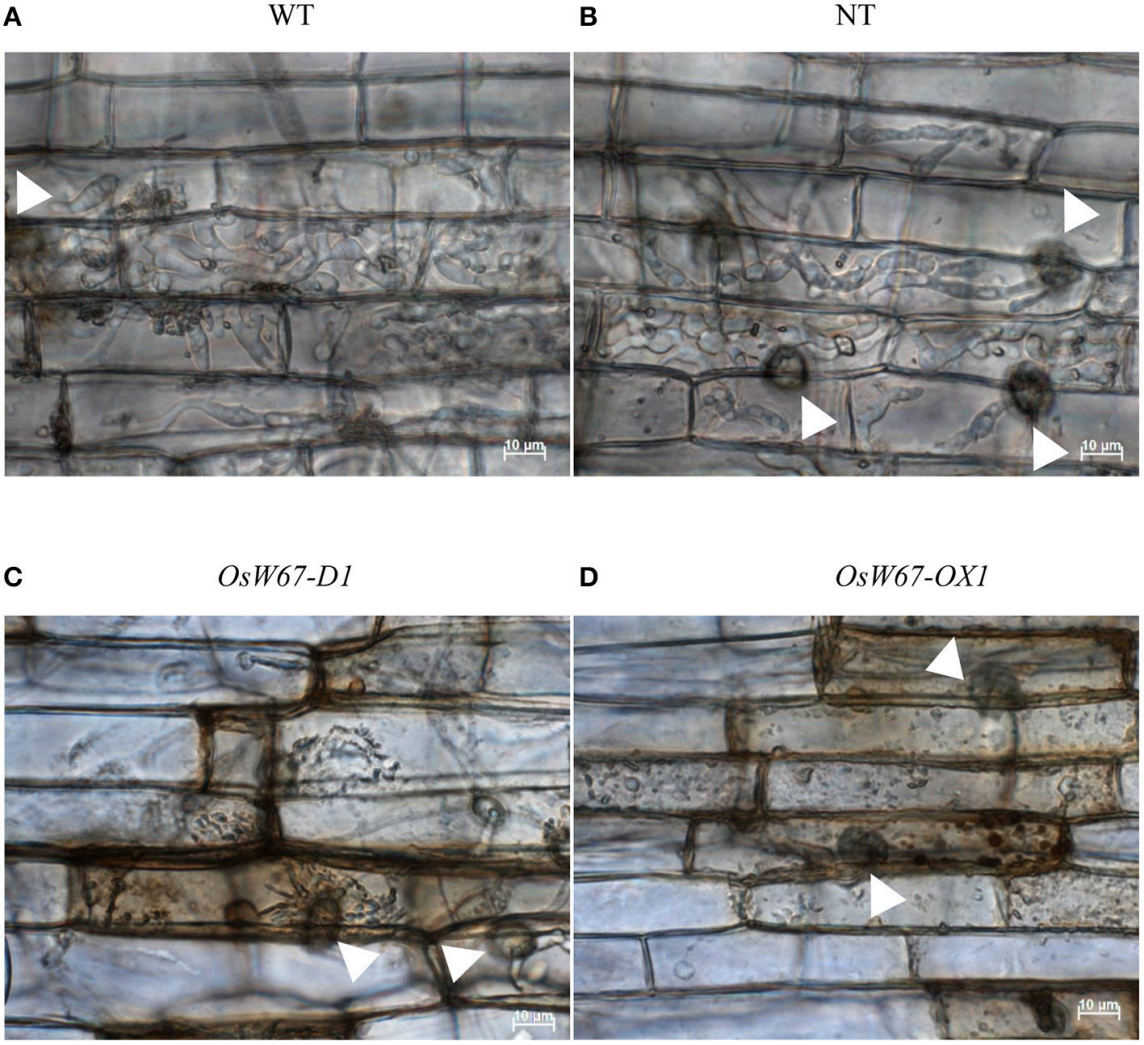
Sheath inoculation assay of *M. oryzae* PO6-6 in *OsWRKY67* activation mutant and overexpression lines. **(A)** Wild type. **(B)** Nontransgene-bearing wild type. **(C)** *OsWRKY67* activation mutant. **(D)** *OsWRKY67* overexpression line. Excised rice sheaths from 5-week-old rice seedlings were inoculated with conidial suspension (2 × 10^4^ conidia m l^−1^). Fungal invasive growth in rice sheath cells was observed at 48 h post infection under light microscopy. Arrow heads indicate appressorium. WT is a wild type control cultivar, Dongjin; NT is a segregant nontransgene-bearing wild type.

A rice leaf sheath infiltration assay was performed to further investigate the role of OsWRKY67 in defense. The *OsW67-D1* and *OsW67-OX1* lines were tested in this experiment for resistance to *M*. *oryzae* PO6-6. Infection hyphae of the controls, wild type and segregated nontransgene-bearing wild type (NT), filled the first-invaded cells and moved to the adjacent cells within 48 h after inoculation, indicating typical susceptible disease symptoms (Figures 4A,B). In contrast, in the *OsW67-D1* and *OsW67-OX1* lines, hypersensitive response (HR)-like cell deaths were observed around the attacked cells. Massive HR-like cell deaths were observed as browning in the penetrated host cells with little or no fungal growth in *OsW67-D1* and *OsW67-OX1* lines (Figures 4C,D). This finding suggests that *OsWRKY67* plays a positive role in the defense response.

### Evaluation of disease resistance of *OsWRKY67* RNAi lines

**Figure 5:**
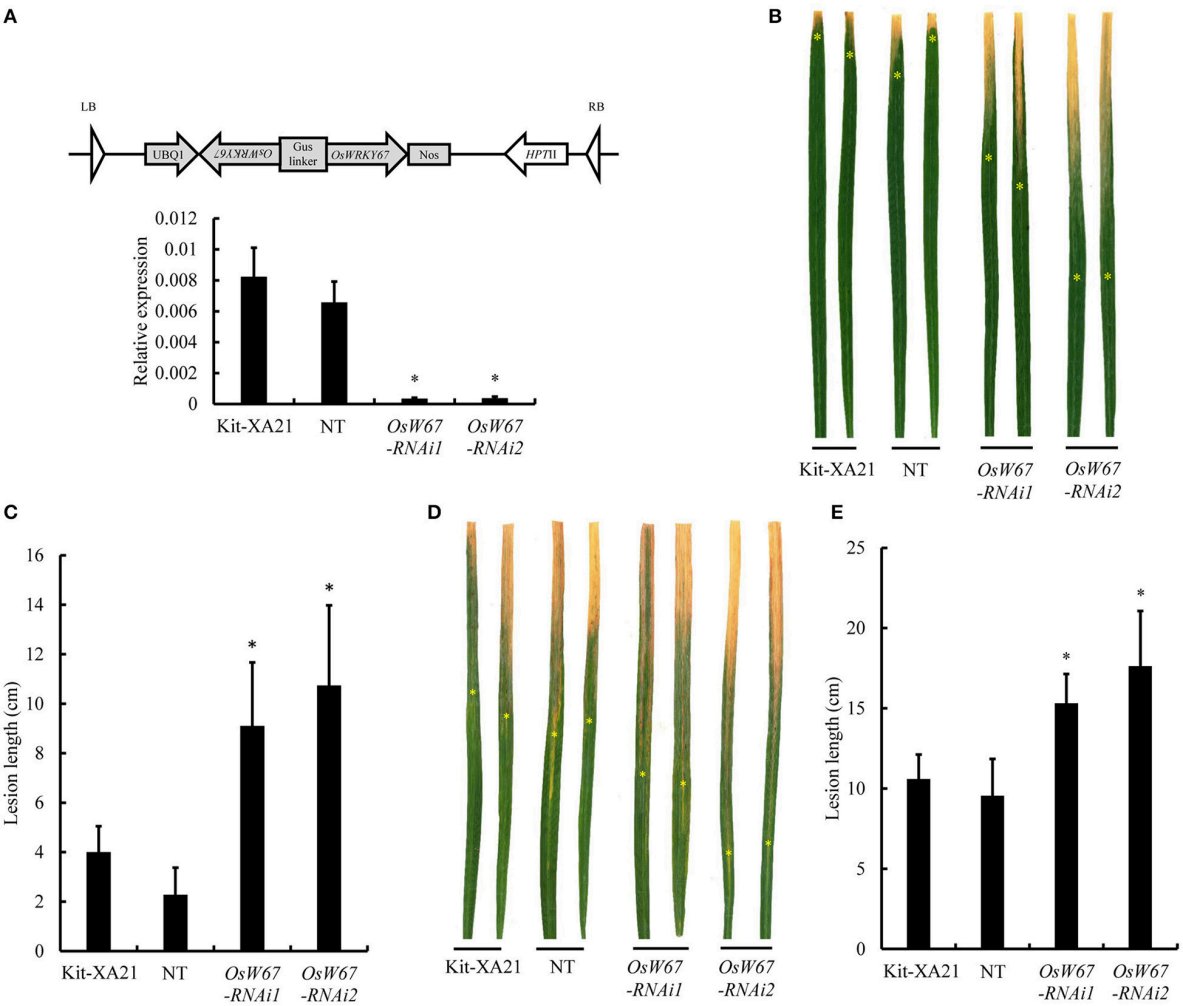
Disease resistance phenotype of *OsWRKY67* RNAi lines to *Xoo* isolates. **(A)** Schematic representation of the *OsWRKY67* RNAi construct. Sense and antisense boxes indicate a unique 359 bp fragment of *OsWRKY67* and antisense sequence (Top). Suppression of *OsWRKY67* transcripts were validated by qPCR. *OsUbq5* is a PCR control. Kit-XA21 is a transformation background genotype; NT is a segregant nontransgene-bearing wild type. **(B)** Representative leaves 12 days after inoculation with *Xoo* PXO99. Yellow asterisks indicate bottoms of lesions. **(C)** Disease lesion lengths measured 12 days after inoculation with *Xoo* PXO99. **(D)** Representative leaves 12 days after inoculation with *Xoo* KXO85. **(E)** Disease lesion lengths measured 12 days after inoculation with *Xoo* KXO85. Data are represented as means ± SD. Asterisks represent statistical significance with Student's *t*-test, *p* < 0.0001.

OsWRKY67 was identified as a component in our XA21 protein interaction network study (Seo et al., 2011). This prompted us to test the role of OsWRKY67 in XA21-mediated resistance, as well as in basal resistance. Therefore, we introduced the *OsWRKY67* RNAi construct (Figure 5A, Top) into the Kitaake line expressing *Xa21* (Kit-XA21) (Park et al., 2010). A significantly reduced expression of *OsWRKY67* was validated by qPCR in two RNAi lines, *OsW67-RNAi1* and *OsW67-RNAi2* (Figure 5A, Bottom). The two independent lines obtained were inoculated with the incompatible *Xoo* PXO99, using the leaf clipping method (Han et al., 2013). The *OsW67-RNAi* lines with *Xa21* exhibited a remarkable increase of disease lesion length (Figures 5B,C). This suggests that OsWRKY67 function is necessary for XA21 resistance. To examine any effect of OsWRKY67 in basal resistance to *Xoo*, we challenged the *OsWRKY67* RNAi lines with a compatible *Xoo* isolate, KXO85. The isolates caused susceptible lesions of 9 and 11 cm in the controls, the segregated nontransgene-bearing plants lacking the RNAi construct (NT) and Kit-XA21, respectively. In this test, *OsW67-RNAi1* and *OsW67-RNAi2* lines developed significantly longer lesions against the *Xoo* isolate, KXO85 (Figures 5D,E). The result indicates that OsWRKY67 function is also required for basal resistance.

**Figure 6:**
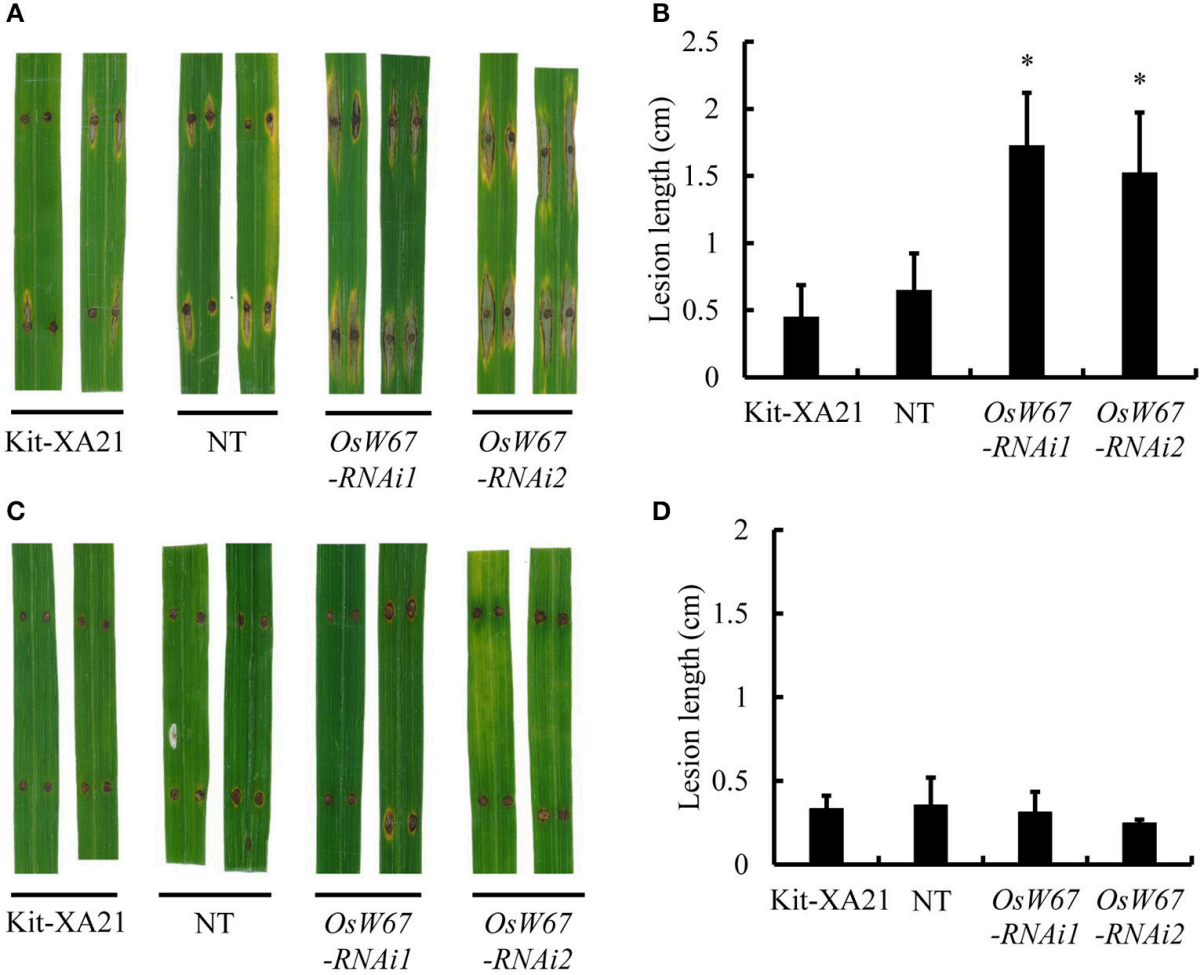
Disease resistance phenotype of *OsWRKY67* RNAi lines to *M. oryzae* isolates. **(A)** Representative leaves 9 days after inoculation with a virulent *M. oryzae*, RO1-1. Kit-XA21 is a transformation background genotype; NT is a segregant nontransgene-bearing wild type. **(B)** Disease lesion lengths measured 9 days after inoculation with a virulent *M. oryzae*, RO1-1. **(C)** Representative leaves 9 days after inoculation with an avirulent *M. oryzae*, PO6-6. **(D)** Disease lesion lengths measured 9 days after inoculation with an avirulent *M. oryzae*, PO6-6. Data are represented as means ± SD. Asterisks represent statistical significance with Student's *t*-test, *p* < 0.0001.

Next, we infected the *OsWRKY67* RNAi lines with the compatible and incompatible *M. oryzae* isolates, RO1-1 and PO6-6, respectively, to the Kitaake cultivar. The *OsWRKY67* RNAi lines displayed wider and longer lesions at 9 DPI to the compatible *M. oryzae* RO1-1, while the controls, Kit-XA21 and nontransgene-bearing plants, developed a limited infected area around the pressed spot (Figures 6A,B). In contrast, the incompatible isolate PO6-6 did not cause any difference in disease reaction among *OsWRKY67* RNAi and controls (Figures 6C,D). These results indicate that OsWRKY67 function is required for basal resistance, but not for resistance to *M. oryzae* in Kitaake mediated by the R gene, a yet-unknown gene.

### Subcellular localization and transcriptional activation ability of OsWRKY67

**Figure 7:**
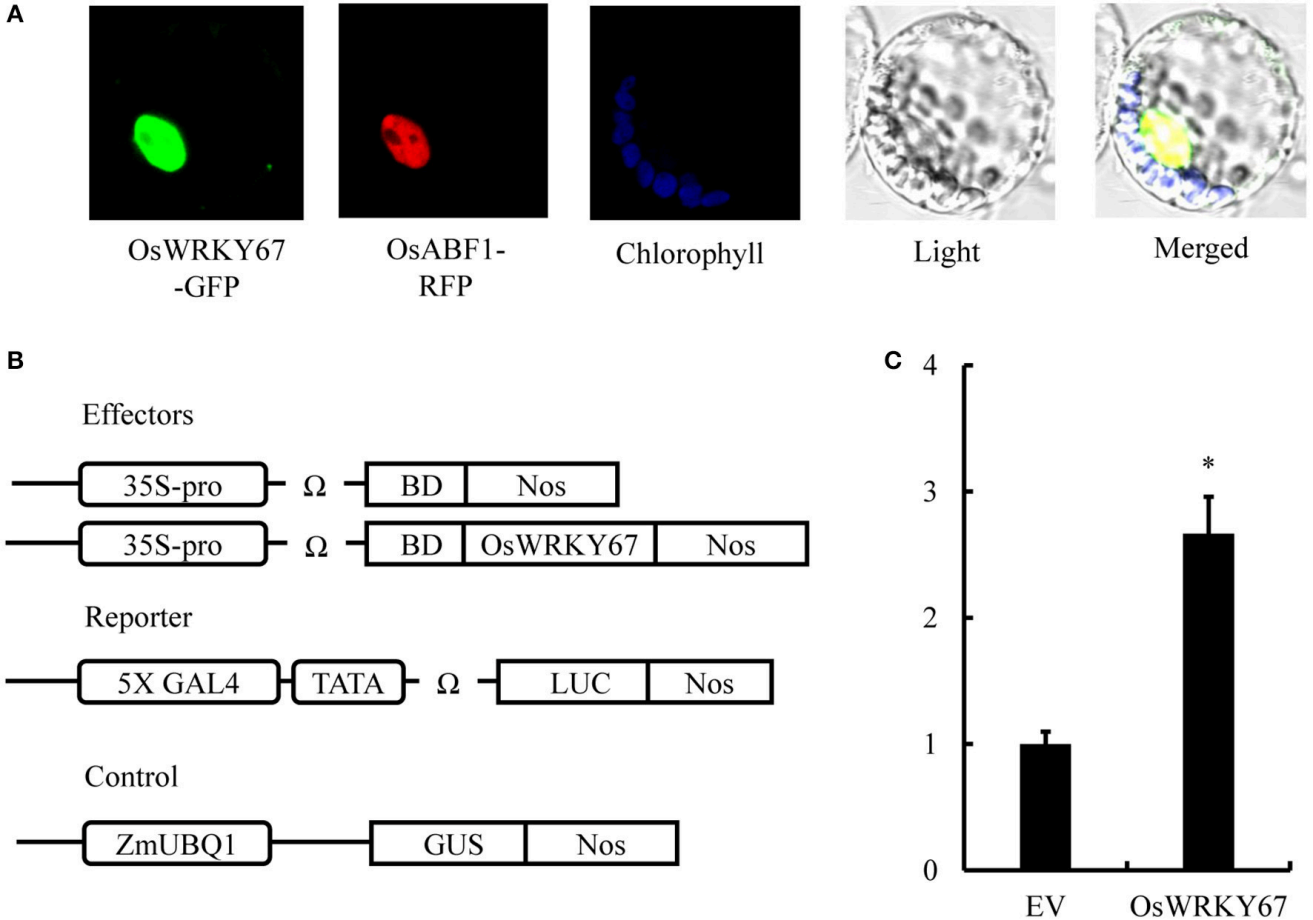
Subcellular localization and transcriptional activity of OsWRKY67. **(A)** Nuclear localization of the OsWRKY67-GFP in maize mesophyll protoplasts. Photographs were taken in the dark field for green fluorescence and in the bright field for cell morphology. OsABF1-RFP is a nuclear marker. Chlorophyll autofluorescence was used as a chloroplast marker. OsWRKY67-GFP signals are green, and nuclear signals are red. **(B)** Schematic representation of constructs used for analysis of GAL4-responsive transcription by OsWRKY67 fused to the GAL4 BD in maize protoplasts. *ZmUBQ1:GUS* was used as an internal control. **(C)** Relative luciferase activities in maize protoplasts after cotransfection with the reporter, effector, and control plasmids. The fold changes were calculated as the LUC/GUS activity ratio. LUC activities were normalized after transfection with the empty vector (EV) control (arbitrarily set at 1). Asterisks represent statistical significance with Student's *t*-test, *p* < 0.0001.

To determine subcellular localization of OsWRKY67, the *OsWRKY67-GFP* fusion construct under the control of the 35S promoter was generated. In a transient expression assay using maize mesophyll protoplasts, the signal of OsWRKY67-GFP was detected exclusively in the nucleus (Figure 7A), as confirmed by their co-localization with the nuclear marker, OsABF1-RFP (Hossain et al., 2010), suggesting that OsWRKY67 is a nuclear localized protein and may thus function as a transcription factor to regulate defense response genes.

We conducted a transcriptional activity assay by transiently expressing *OsWRKY67* in maize protoplasts to determine whether OsWRKY67 is an activator or a suppressor. The reporter plasmid carrying 5X GAL4, a minimal TATA, the Ω sequence, and the firefly *LUC* gene was co-introduced into maize protoplasts with an effector plasmid carrying *OsWRKY67* and the GAL4 BD domain driven by 35S promoter and the Ω sequence (Figure 7B). The significant induction of LUC activity compared to 35S-driven GAL4-DB reveals that OsWRKY67 is a transcriptional activator (Figure 7C). A yeast transformant containing the OsWRKY67-BD bait construct grew consistently well on SD selection media, and showed β-galactosidase activity in an X-gal filter assay (Figure S2).

### Expression of PR genes in *OsWRKY67* overexpression lines

**Figure 8:**
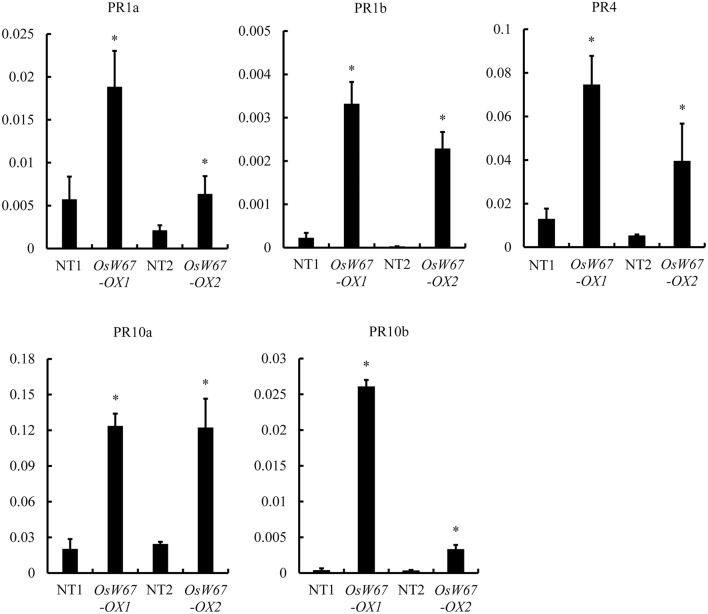
Expression of PR genes in *OsWRKY67* overexpression lines, *OsW67-OX1* and *OsW67-OX2*. The y-axis on the graphs indicates the relative expression. *OsUbq5* served as an internal control. NT1 is a segregant nontransgene-bearing wild type of *OsW67-OX1*; NT2 is a segregant nontransgene-bearing wild type of *OsW67-OX2*. Asterisks represent statistical significance with Student's *t*-test, *p* < 0.0001.

To obtain molecular understanding of *OsWRKY67*-mediated resistance, we examined expression of PR genes in two *OsWRKY67* overexpression lines, *OsW67-OX1* and *OsW67-OX2*, and their respective controls, the segregated nontransgene-bearing wild types in their next generation. In qPCR analysis, all tested PR genes, *PR1a, PR1b, PR4, PR10a*, and *PR10b* (Wang et al., 2015), were upregulated in the *OsWRKY67* overexpression lines compared with their control lines (Figure 8), which is consistent with their enhanced disease resistance.

## Discussion

### OsWRKY67 is a positive regulator of the defense response in rice

On the basis of the previous finding of upregulation of *OsWRKY67* in response to pathogens (Ryu et al., 2006), we characterized here, a function of OsWRKY67 in the defense response using its gain- and loss-of-function rice lines. The T-DNA activation tagging lines, *OsW67-D1* and *OsW67-D2*, and the *OsWRKY67* overexpression lines, all enhanced disease resistance to pathogens *M. oryzae* and *Xoo* (Figures 1, 3, 4). The rapid production of ROS plays a pivotal role in disease resistance response (Fujita et al., 2006; Park et al., 2012). In this regard, we found that the *OsW67-D1* and *OsW67-D2* lines accumulated ROS rapidly and highly in response to elicitor treatments (Figure 2). In contrast, the *OsWRKY67* RNAi lines created in the background carrying the *Xa21* gene reduced both basal and XA21-mediated disease resistance to pathogens (Figures 5, 6). These results all clearly demonstrated that OsWRKY67 positively regulates disease resistance to the rice pathogens tested (*M. oryzae* and *Xoo*). OsWRKY67 is present in the nucleus and appeared to function as an activator (Figure 7), which suggests that it is a transcriptional activator. Consistently, we found an upregulation of PR genes in *OsWRKY67* overexpression lines (Figure 8). Therefore, it is likely that OsWRKY67 can activate certain downstream target genes, which subsequently upregulates the defense response.

### OsWRKY67 is necessary for XA21-mediated resistance

Previously, in order to elucidate stress response signaling networks, an interactome of 100 proteins was established by yeast-two-hybrid assays around key regulators of the rice biotic and abiotic stress responses (Seo et al., 2011); XA21 stands as a key hub protein tested to construct the XA21 interactome network. Eight unique XA21 binding proteins (XBs) were identified and further used to screen XB interacting proteins (XBIPs). Among the eight XBs, five play an important role in regulating the rice defense response against *Xoo:* an ATPase (XB24), an E3 ubiquitin ligase (XB3), a PP2C phosphatase (XB15), OsWRKY62 (XB10), and an ankyrin-repeat protein (XB25). In the branch of a negative regulator OsWRKY62, three other OsWRKYs were found; OsWRKY28 and OsWRKY76 are negative regulators, whereas OsWRKY71 is a positive regulator in the defense response. The involvement of OsWRKY67, in addition to OsWRKY28, OsWRKY62, and OsWRKY76, in the XA21-interactome network raises the possibility that it may be necessary for XA21-mediated resistance. Indeed, the *OsWRKY67* RNAi lines compromised XA21-mediated resistance (Figures 5B,C), suggesting its important role in XA21-mediated resistance. OsWRKY67 was found to interact with XB21, which is similar to p23, a protein that modulates HSP90-mediated folding of key molecules involved in various signal transduction pathways (Seo et al., 2011). It would be valuable in the future to elucidate mechanisms by which OsWRKY67 regulates XA21-mediated resistance.

### Manipulation of OsWRKY67 has the potential to enhance broad-spectrum disease resistance in rice

In previous studies, overexpression of *OsWRKY45* elevated disease resistance to biotrophic and hemibiotrophic pathogens (Shimono et al., 2007, 2012). When *OsWRKY45* was introduced to rice under the control of several promoters to enhance its expression, defense responses were enhanced but plant growth and yield were compromised in strong expressors (Goto et al., 2014). Similarly, we observed a dwarfed phenotype due to growth retardation from certain *OsWRKY67* overexpression lines, in conjunction with increased levels of OsWRKY67 protein (Figure 3B). Indeed, there is a documented trade-off between disease resistance and plant growth and fitness; more than 60 genes have been manipulated to increase crop resistance, such as those that code for protein receptors, signaling factors, transcription factors, and defense response genes, and detrimental effects on plant growth were found in approximately 80% of the cases (Delteil et al., 2010). For instance, the lesion mimic phenotype is a typical drawback related to the constitutive expression of PR genes to activate defense systems. Growth retardation and reduced fitness are frequently found when transcription factors involved in defense are overexpressed; overexpression of *OsNAC6* limited plant height, and overexpression of *OsWRKY31* attenuated root growth (Nakashima et al., 2007; Zhang et al., 2008). Until now, at least 12 OsWRKY genes have been manipulated in an attempt to improve traits against pathogen infection in rice, but none have been declared optimal due to weak resistance or compromised agronomic traits (Delteil et al., 2010). However, our current work shows that T-DNA activation lines (*OsW67-D1* and *OsW67-D2*), and weak overexpression lines (*OsW67-OX1*) enhanced resistance without significant growth retardation (Figures 1, 3). This suggests that OsWRKY67 is a promising target protein candidate for genetic manipulation in rice, in which well-regulated increases in the expression of *OsWRKY67* can provide broad-spectrum resistance without a growth penalty.

## Author contributions

KV, C-YK, TH, S-KL, and GS performed the experiments; KV, C-YK, Y-SS, S-WL, G-LW, and J-SJ analyzed the data; and KV, C-YK, Y-SS, S-WL, G-LW, and J-SJ wrote the paper. All authors read and approved the final manuscript.

### Conflict of interest statement

The authors declare that the research was conducted in the absence of any commercial or financial relationships that could be construed as a potential conflict of interest.

## Supplementary material

Figure S1 Evaluation of OsWRKY67 phosphorylation. Total protein extracted from *OsWRKY67* overexpressing lines were incubated with (+) or without (–) lambda phosphatase, and separated by SDS-PAGE. OsWRKY67-Myc protein was detected by Western blotting using anti-c-Myc antibody.

Figure S2 Transcriptional activity assay of OsWRKY67 in yeast PBN204 strain with *lacZ, URA3*, and *ADE2* as reporters. OsWRKY67 was fused to the GAL4 DNA-binding domain of pGBKT7, which was then introduced into yeast (#1). Transformed yeast cells were plated onto a selective medium lacking Leu and Trp (SD-LW) or a selective medium also lacking Ade (SD-LWA) or Ura (SD-LWU). Positive (+) control had yeast transformed with the P53 bait plasmid and the Tag prey plasmid. Negative (–) control had yeast transformed with the parental bait vector (pGBKT7) and the prey vector (pACT2).

Table S1 Primers used in this study.
