# Supplementary figures and images for: OsWRKY67 Plays a Positive Role in Basal and XA21-Mediated Resistance in Rice
Source: Front Plant Sci. 2018 Jan 11;8:2220. doi: 10.3389/fpls.2017.02220 (PMC5769460; doi:10.3389/fpls.2017.02220)

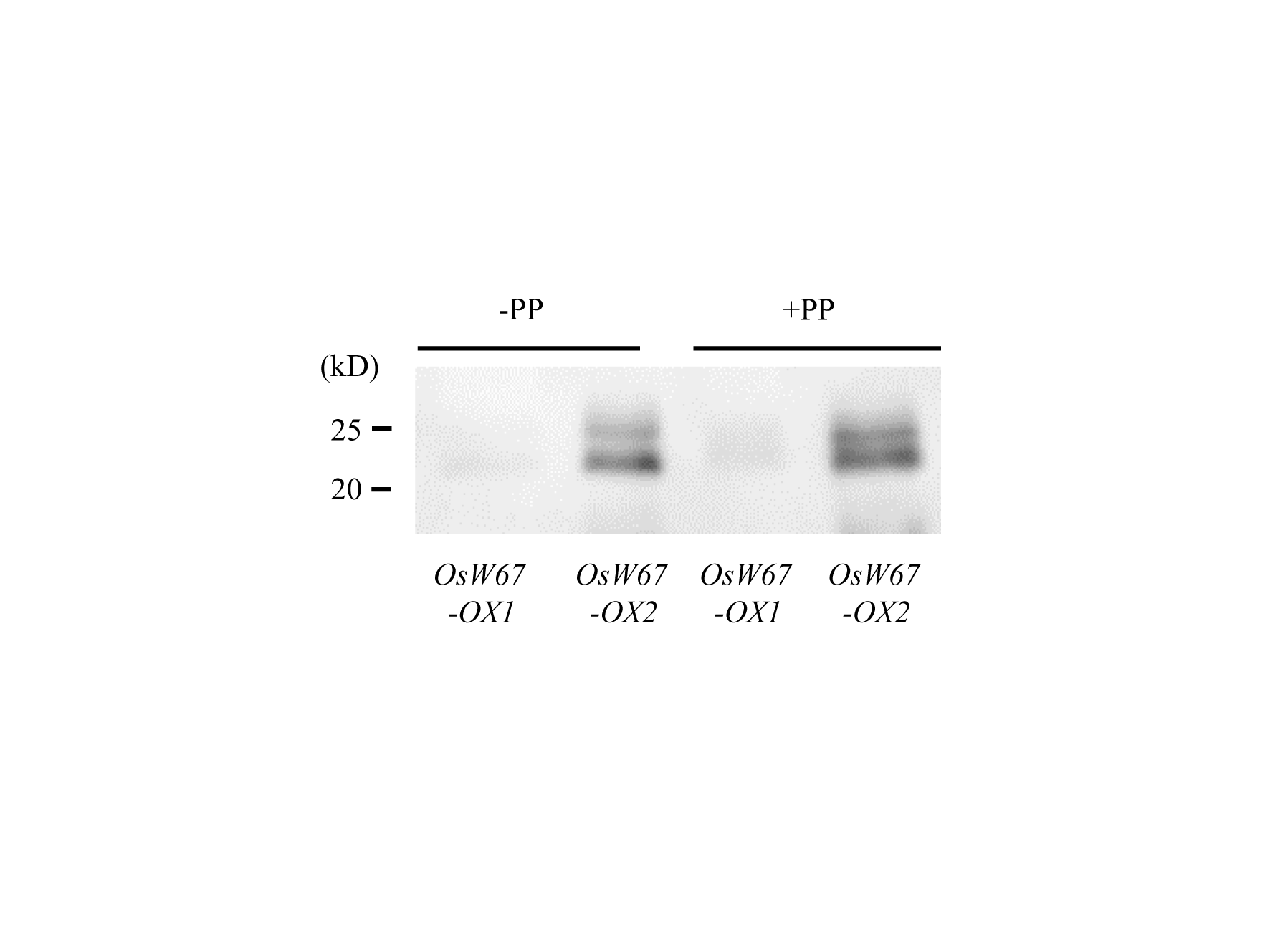

Supplement: Figure S1 — Evaluation of OsWRKY67 phosphorylation. Total protein extracted from OsWRKY67 overexpressing lines were incubated with (+) or without (–) lambda phosphatase, and separated by SDS-PAGE. OsWRKY67-Myc protein was detected by Western blotting using anti-c-Myc antibody. [file Image1.TIF]

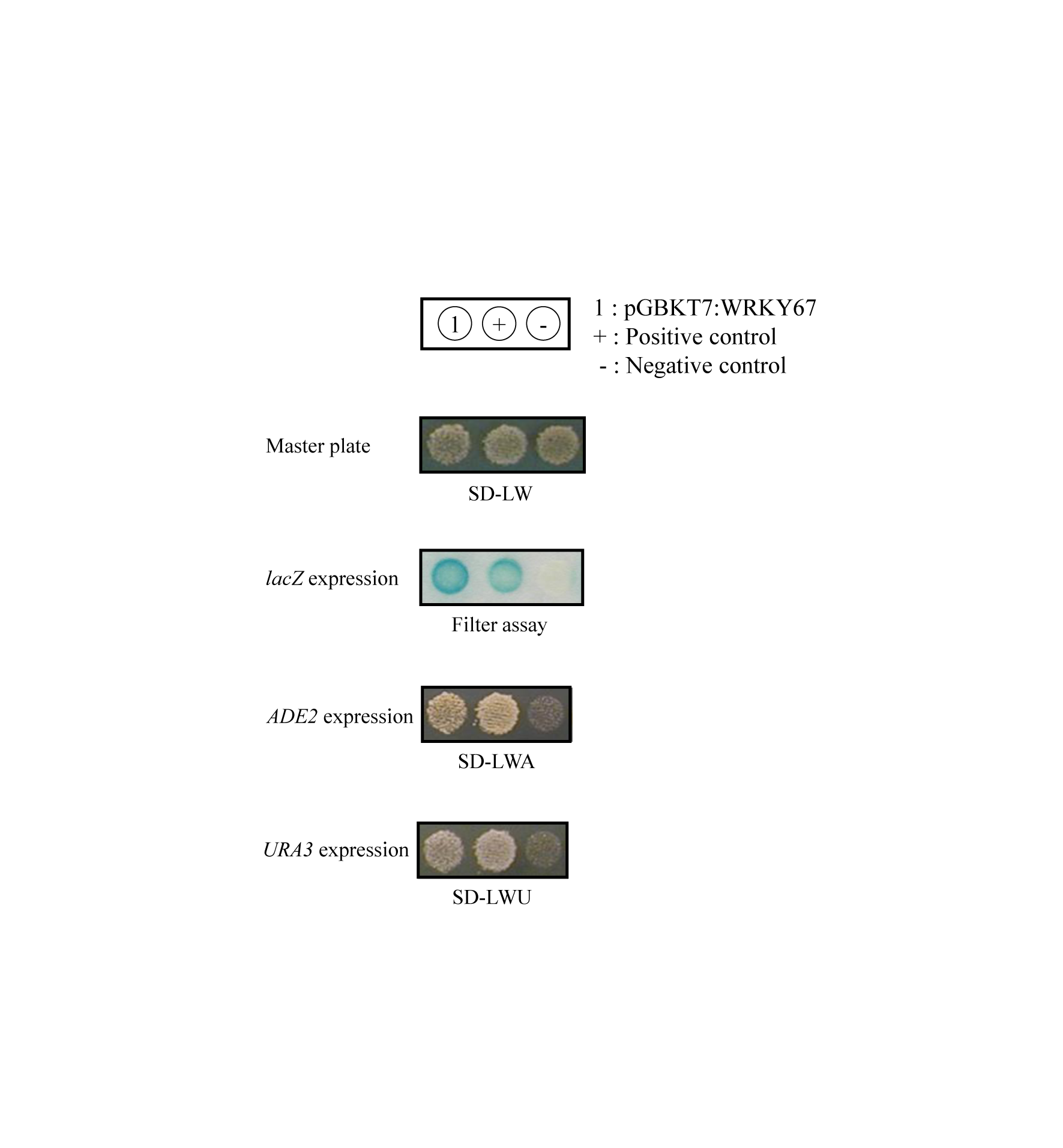

Supplement: Figure S2 — Transcriptional activity assay of OsWRKY67 in yeast PBN204 strain with lacZ, URA3, and ADE2 as reporters. OsWRKY67 was fused to the GAL4 DNA-binding domain of pGBKT7, which was then introduced into yeast (#1). Transformed yeast cells were plated onto a selective medium lacking Leu and Trp (SD-LW) or a selective medium also lacking Ade (SD-LWA) or Ura (SD-LWU). Positive (+) control had yeast transformed with the P53 bait plasmid and the Tag prey plasmid. Negative (–) control had yeast transformed with the parental bait vector (pGBKT7) and the prey vector (pACT2). [file Image2.TIF]
