# Supplementary material for: OsWRKY67 Plays a Positive Role in Basal and XA21-Mediated Resistance in Rice
Source: Front Plant Sci. 2018 Jan 11;8:2220. doi: 10.3389/fpls.2017.02220 (PMC5769460; doi:10.3389/fpls.2017.02220)
Supplement: Table S1 — Primers used in this study. [file Table1.DOCX]

Table S1. Primers used in this study.

| **Primer** | **Sequence** | **Experiment** |
| --- | --- | --- |
| OsW67-D1-F | CGCTGCACCCATAGATGCACAAAGCCATG | Genotyping |
| OsW67-D1-R | AGGCAACAGCTGATGTGTGAGGAACGGGC | Genotyping |
| OsW67-D2-F | GGCTGTCCCATCGGAGCGTCATTCGGAC | Genotyping |
| OsW67-D2-R | TTGGGCACGGATATCCACGGCTGCACTT | Genotyping |
| 2715LB | ATCTTGAACGATAGCCTTTCCTTTATCG | Genotyping |
| OsW67-F | ATGAGGTACGAGAGCGAG | Overexpression |
| OsW67-R | TCAGAAGAGCAGCGAGCCGCC | Overexpression |
| OsW67-RT-F | CTTCAGCAACTCCTACTCCTACT | qPCR |
| OsW67-RT-R | TCTCGTACCTCATCATAGTGTTT | qPCR |
| OsW67-RNAi-F | CACCCCATGAAAGGGGATGAGGAG | RNAi |
| OsW67-RNAi-R | GGTGGTCCTCCCGGTCTCTC | RNAi |
| PR1a-RT-F | AGAACTCGGCGCAGGACTTC | qPCR |
| PR1a-RT-R | GGAGCCCCAGAAGATGTTCTC | qPCR |
| PR1b-RT-F | GAGGTATCCAAGCTGGCCATT | qPCR |
| PR1b-RT-R | CTTCTCTGGCTGGCGTAGTTC | qPCR |
| PR10a-RT-F | GCACCATCTACACCATGAAGC | qPCR |
| PR10a-RT-R | TCGAGTGTGACTTGAGCTTCC | qPCR |
| PR10b-RT-F | CTGTGGAAGGTCTGCTTGGAC | qPCR |
| PR10b-RT-R | ACACCTCAACCTTTAGCACGT | qPCR |
| PR4-RT-F | TTGTCTTGGCGCCAGAAGTAT | qPCR |
| PR4-RT-R | CCATTGCTGCATTGGTCAACA | qPCR |
| OsW67-YF | CGGAATTCGCG GCTTCCGTAGGACTG | Yeast assay |
| OsW67-YR | CGGGATCCTCAGAAGAGCAGCGAGCCG | Yeast assay |
| OsW67-SmalI-F | CCCCCGGGATGGCGGCTTCCGTAGGACT | Transcriptional assay |
| OsW67-SalI-R | GCGTCGACTCAGAAGAGCAGCGAGCCGC | Transcriptional assay |
